# Changes in undergraduate student alcohol consumption as they progress through university

**DOI:** 10.1186/1471-2458-8-163

**Published:** 2008-05-19

**Authors:** Bridgette M Bewick, Brendan Mulhern, Michael Barkham, Karen Trusler, Andrew J Hill, William B Stiles

**Affiliations:** 1 Leeds Institute of Health Sciences, University of Leeds, UK; 2 Centre for Psychological Services Research, University of Sheffield, UK; 3 Department of Psychology, Miami University, USA

## Abstract

**Background:**

Unhealthy alcohol use amongst university students is a major public health concern. Although previous studies suggest a raised level of consumption amongst the UK student population there is little consistent information available about the pattern of alcohol consumption as they progress through university. The aim of the current research was to describe drinking patterns of UK full-time undergraduate students as they progress through their degree course.

**Method:**

Data were collected over three years from 5895 undergraduate students who began their studies in either 2000 or 2001. Longitudinal data (i.e. Years 1–3) were available from 225 students. The remaining 5670 students all responded to at least one of the three surveys (Year 1 n = 2843; Year 2 n = 2219; Year 3 n = 1805).

**Results:**

Students reported consuming significantly more units of alcohol per week at Year 1 than at Years 2 or 3 of their degree. Male students reported a higher consumption of units of alcohol than their female peers. When alcohol intake was classified using the Royal College of Physicians guidelines [1] there was no difference between male and females students in terms of the percentage exceeding recommended limits. Compared to those who were low level consumers students who reported drinking above low levels at Year 1 had at least 10 times the odds of continuing to consume above low levels at year 3. Students who reported higher levels of drinking were more likely to report that alcohol had a negative impact on their studies, finances and physical health. Consistent with the reduction in units over time students reported lower levels of negative impact during Year 3 when compared to Year 1.

**Conclusion:**

The current findings suggest that student alcohol consumption declines over their undergraduate studies; however weekly levels of consumption at Year 3 remain high for a substantial number of students. The persistence of high levels of consumption in a large population of students suggests the need for effective preventative and treatment interventions for all year groups.

## Background

Heavy alcohol intake amongst the student population has implications for the individual, educational institutions, and wider society [2-4]. Students have been reported to drink at higher levels than non-student peers [5-9], making this an issue of public health concern given the negative social and health consequences of heavy intake [10] and the link with other unhealthy behaviours (e.g. cigarette smoking [11] and recreational drug use [12]).

Across the world it has been reported that university students' levels of alcohol consumption are higher than that of their non-university peers [5,13,14]. Within the United Kingdom, a review of studies measuring undergraduate drinking concluded that 52% of males and 43% of females reported drinking above the recommended limits of 21 units per week for men and 14 units per week for women [15]. The comparable figures for 16–24 year olds in the general population are 37% and 33% respectively [16]. Furthermore, 15% of a UK sample of 3075 students drank at hazardous drinking levels of 51 or more units per week for men and 36 or more units per week for women (within the study 1 unit (10 ml ethanol) was taken as 1/2 pint beer/one glass wine/one measure spirits) [17].

The alcohol consumption of university (i.e. college) students in the US has been extensively studied [4,18] and it is generally agreed that, while the trajectories of groups of students differ during their time at university [19-21], the overall trend in consumption is one of increase from high school to university, followed by a decline post-graduation. In contrast with their North American peers, the drinking patterns of students from the UK, and Europe in general, have been less extensively investigated [22]. Unlike in the US, where the minimum purchase age is 21 years, United Kingdom students can legally purchase alcohol from the age of 18, thereby creating a different context for alcohol consumption. Despite research consistently highlighting high consumption levels within the UK student population [15] there is uncertainty regarding whether/how these alcohol consumption patterns change over academic stay.

The majority of UK studies that have considered year of study have utilised a cross-sectional approach, restricted their samples to medical and/or dental students and have shown contradictory patterns [8,23]. The one available longitudinal study of alcohol consumption was also complicated by differences between medical and dental students [24]. There was no overall change in mean alcohol consumption among dental students but an increase among medical students, from second year to final year. In addition, the percentage of dental students drinking more than the recommended limits decreased while it increased in medical students. Academic performance of medical students at UK universities may be adversely affected by heavy alcohol intake [7,25]. However, it is unclear what students think about how their alcohol consumption may be affecting their studies.

As levels of alcohol intake increase, so does the prevalence of a variety of risky behaviours, including unsafe sexual activity [4,26], behaviour leading to injury [27,28] and damage to property [29], violence [30] and illegal behaviour [28]. Increased frequency of injury and assault [31] inevitably leads to increased strain on care and emergency services, and links between alcohol consumption and hospital admissions are well established [32]. Heavy alcohol intake has also been linked to depression and liver damage [33]. In the city where the current study took place, it is estimated that alcohol misuse costs 275 million pounds per year, the majority of which is spent on policing, repairing damage and injury and alcohol-related illness treatment [34].

The present investigation aimed to examine the patterns of alcohol consumption of two cohorts of students over the course of their undergraduate education at a university in the UK, using both cross-sectional and opportunistic longitudinal study designs. It also investigated student perceptions of the consequences of their alcohol consumption.

## Method

### Data set

Data were drawn from the UNIversity Quality of Life and Learning (UNIQoLL) dataset collected at a city-based University in the UK [35]. UNIQoLL was series of cross-sectional surveys examining students' perceptions of university life that covered a variety of issues including mental health, alcohol consumption and the perceived effect of alcohol intake on aspects of student life.

Pen and paper questionnaires were delivered to students via their university department. Departments were asked to distribute a copy of the questionnaire to all undergraduate full-time students. The mode of delivery varied between departments and included: distributing questionnaires in lectures, asking students to collect questionnaires from a designated point, posting a questionnaire to students via the internal mail. Students could also request a copy of the questionnaire directly from the UNIQoLL project team; contact details were provided on promotional posters that were displayed prominently throughout the University.

The project ran from 2000 until 2006 with students being asked to complete a survey at seven time points over their three undergraduate years. On each survey students were asked to provide their student identification number. Where valid student identification numbers were available student responses could be linked across time if students responded to more than one survey. Questions pertaining to alcohol consumption were asked during the spring of semester two of each academic year. Where students responded to all three surveys the linking process provided an opportunistic longitudinal dataset for examining changes in alcohol consumption. Where students completed less than three surveys their data were included in the cross-sectional dataset. This dataset included participants who had responded to only one or two of the three surveys.

### Survey questionnaire

#### Alcohol consumption and attitude measures

Students were asked to record confidentially how many units of alcohol they drank per week, on average (1 unit = 10 ml ethanol). Students were advised that one unit was approximately 1/2 pint of lager, 1 small glass of wine or 1 measure of spirits [36].

Attitudes towards alcohol consumption were assessed by four questions. Students were asked 'To what extent do you believe that your alcohol consumption is having a negative impact on your (i) studies, (ii) finances, (iii) personal life, and (iv) physical health'. Each question was answered on a 5-point Likert scale (1 = definitely not, 2 = probably not, 3 = not sure, 4 = probably, 5 = definitely).

#### Classification of level health risk according to reported alcohol consumption

Current UK government guidelines recommend no more than 4 units of alcohol a day for men and 3 units of alcohol per day for women suggesting upper limits of 28 and 21 units of alcohol per week (u/w) for men and women respectively [36]. These guidelines represent a 33% increase for men and a 50% increase for women on the previous recommended limits [37]. The current recommendations have been criticised, however, by Edwards [38], and the Royal College of Physicians [1] and the British Medical Association [39] have advised men and women to follow the earlier limits of less than 21 and 14 u/w, respectively.

For the present study, weekly alcohol consumption was classified into the following four categories: (1) 'low level drinking' (Low; male 0–21 u/w, female 0–14 u/w) [1,39], (2) 'medium level drinking' (Medium; male 21–28 u/w, female 14–21 u/w) [36], (3) 'high level drinking' (High; male 28–50 u/w, female 31–35 u/w) [30a], and (4) 'Very high level drinking' (Very high; male 50+ u/w, female 35+ u/w) [40]. These classifications were chosen to allow comparisons with previous research that has used both sets of guidelines. They also relate directly to the 'sensible limits' (corresponds to 'low level drinking') and 'hazardous drinking (corresponds to 'very high level drinking') cut points that are currently used within UK governmental reports [40].

### Participants

At each time-point approximately 20% of registered full-time undergraduate students returned a completed survey (year 1 n = 3068, 17%; year 2 n = 2444, 20%; year 3 n = 2030, 20%). In total, 5895 (34%) students who started their degree in either 2000 or 2001 completed at least one of the three second-semester surveys during their three year undergraduate programme. Of these, 225 students provided data for all of the three academic years (i.e. an opportunistic longitudinal sample). Of the remaining cross-sectional sample (n = 5670), 674 students provided data at both year 1 and year 2, 230 participants provided data at both year 1 and year 3, and 293 at year 2 and year 3.

Overall, 52% of participants were female, 38% were male and in 10% sex was unknown. Eighty four percent were aged under 21 on entry to university and 84% were students from the United Kingdom. The mean age at year 1 was 19.49 yrs (sd = 2.40). The majority of participants (80%) were white/white British. Participants were drawn from all university faculties with the largest percentages from Biological Sciences (15%), Joint Honours (15%), and Faculty of Arts (13%). No statistical differences were observed between the longitudinal and cross-sectional samples in terms of sex (Mann-Whitney U = 534592.5, p > 0.05), age at year 1 (t = 1.56, df = 2839. p = 0.12), age at year 2 (t = 1.70, df = 2243, p = 0.10) or alcohol consumption (Y1 t = 0.31, df = 3.66, p = 0.76; Y2 t = -0.32, df = 2442, p = 0.75' Y3 t = -0.75, df = 300.01 (equal variance not assumed), p = 0.46). A small (longitudinal mean 21.13 SD 2.13; cross-sectional mean 21.70, SD = 2.67) but statistical difference of age was observed between the two samples at year 3 (t = -3.66, df = 1866, p < 0.01). The demographics of participants were similar to those of the wider student population.

### Data analysis

The means and standard deviations reported in the text and tables of this article are based on the raw data. However, alcohol consumption (in units/week) for both the longitudinal and cross-sectional samples were positively skewed, and therefore all inferential analyses used square-root transformations of the data. To analyse the longitudinal sample, repeated measures ANOVA was employed to investigate the change in units of alcohol consumed over time. Changes, within the longitudinal sample, in perceptions of the negative impact of alcohol were analysed using repeated measures ANOVA. Year differences in units of alcohol consumed were investigated within the cross-sectional sample using ANOVA. For the purposes of this analysis the independent variable was time (i.e. Year) and alcohol consumed (in units) was the dependent variable.

For both the longitudinal and the cross-sectional samples the percentage of students drinking above the low consumption level was calculated at each time-point. Chi-squared analysis was used to investigate differences in the percentage of male and female participants exceeding the sensible limits guidelines. Independent t-tests were used to compare reported weekly units between males and females each time point.

For the longitudinal sample, odds ratios were calculated to investigate the likelihood of those students drinking above low levels at Year 1 continuing to report this level of drinking at Years 2 and 3. Similarly, odds ratios were calculated to investigate the likelihood of year 2 and year 3 students drinking above low levels compared to students in year 1.

Within both the longitudinal and cross-sectional samples the percentage of students who reported that alcohol was having a negative impact (i.e. definitely or probably) on each of the four life areas were calculated, and odds ratios were used to determine the likelihood of those students drinking above low levels reporting a negative impact compared to their peers who reported little or no negative impact.

## Results

### Units of alcohol consumed per week

Taken across samples and time points, some 90% of the sample measured at each time point indicated that they consumed at least 1 u/w. Repeated measures ANOVA identified a significant decrease in alcohol consumption for students in the longitudinal sample over time (Huynh-Feldt adjustment F = 38.17, df = 1.97, 440, p < 0.01) but no sex by time interaction (Huynh-Feldt adjustment F = 1.173, df = 1.97, 440, p > 0.05). A priori mean comparison tests showed significant differences (p < 0.01) between each time point with consumption consistently decreasing overtime (see Table 1).

**Table 1:** Units per week and effect sizes between genders for both longitudinal and cross-sectional

	Longitudinal								Cross-sectional							
	Total		Male		Female				Total		Male		Female			
	M	SD	M	SD	M	SD	*t*	df	M	SD	M	SD	M	SD	*t*	df

Year 1	19.11	13.94	24.69	15.60	16.03	11.91	-3.38*	223	18.94	14.45	24.04	16.16	15.49	11.89	-12.22*	2097^a^
Year 2	15.49	12.53	20.21	12.97	12.89	11.52	-3.80*	223	16.06	13.36	21.09	15.60	12.65	10.09	-10.56*	1420^a^
Year 3	12.62	9.99	16.34	12.14	10.57	7.91	-2.72*	128^a^	13.89	12.52	18.42	14.17	10.71	9.79	-10.88*	1296^a^

^a ^= Levene's test p < 0.05 test statistics reported do not assume equal variances

*p < 0.01

Students in the cross-sectional sample also reported a significant difference in their alcohol consumption between year groups (F = 71.64, df = 2, 6864, p < 0.01). A priori mean comparison tests showed significant differences (p < 0.01) between each year group with consumption decreasing from Year 1 to Years 2 and 3 (see Table 1).

### Classification of health risk by level of alcohol consumption

At Year 1, 50% (n = 1297) of students in the cross-sectional sample reported consumption levels that exceeded the 21/14 u/w low level threshold. The corresponding figures for Years 2 and 3 were 42% (n = 804) and 32% (n = 502) respectively (Table 2). At Year 1, 53% (n = 120) of students in the longitudinal sample reported consumption levels that exceeded the 21/14 u/w low level threshold. This proportion reduced to 38% (n = 85) during Year 2 and 30% (n = 68) at Year 3.

**Table 2:** Classification of alcohol consumption by year of study by sample and gender

	Cross-sectional		Longitudinal		Cross-sectional		Longitudinal		Cross-sectional		Longitudinal	
	Male		Male		Female		Female		Total		Total	
	N	(%)	N	(%)	N	(%)	N	(%)	N	(%)	N	(%)
**1st years**												
Low level drinking (low)	553	(51)	38	(48)	746	(49)	67	(46)	1299	(50)	105	(47)
Medium level drinking (medium)	91	(8)	7	(9)	420	(28)	40	(28)	511	(20)	47	(21)
High level drinking (high)	313	(29)	28	(35)	225	(15)	28	(19)	538	(21)	56	(25)
Very high level drinking (very high)	130	(12)	7	(9)	118	(8)	10	(7)	248	(10)	17	(8)
												

**2nd years**												
Low level drinking (low)	442	(57)	48	(60)	682	(59)	92	(63)	1124	(58)	140	(62)
Medium level drinking (medium)	67	(9)	8	(10)	302	(26)	26	(18)	369	(19)	34	(15)
High level drinking (high)	203	(26)	21	(26)	121	(11)	19	(13)	324	(17)	40	(18)
Very high level drinking (very high)	63	(8)	3	(4)	48	(4)	8	(6)	111	(6)	11	(5)
												

**3rd years**												
Low level drinking (low)	443	(67)	59	(74)	629	(69)	98	(68)	1072	(68)	157	(70)
Medium level drinking (medium)	48	(7)	4	(5)	194	(21)	36	(25)	242	(15)	40	(18)
High level drinking (high)	137	(21)	16	(20)	62	(7)	10	(7)	199	(13)	26	(12)
Very high level drinking (very high)	34	(5)	1	(1)	27	(3)	1	(1)	61	(4)	2	(1)

Male Low = 0–21 u/w, Medium = 21–28 u/w, High = 28–50 u/w, Very high = 50+ u/w

Female Low = 0–14 u/w, Medium = 14–21 u/w, High = 21–35 u/w, Very high = 35+ u/w

There was a significant (p < 0.01) difference in alcohol consumption between male and female participants at each time point with males consistently reporting consuming more units per week (see Table 1). However, no differences were found in the proportions of males and females exceeding the low level threshold (see Table 2). Chi-squared analysis revealed no significant differences in the percentages of men and women exceeding the low level threshold in either the cross-sectional (Y1 χ^2 ^= 0.52, df = 1, p = 0.47; Y2 χ^2 ^= 0.86, df = 1, p = 0.36; Y3 χ^2 ^= 0.74, df = 1, p = 0.39) or the longitudinal sample (Y1 χ^2 ^= 0.04, df = 1, p = 0.85; Y2 χ^2 ^= 0.26, df = 1, p = 0.61; Y3 χ^2 ^= 0.93, df = 1, p = 0.34).

Those students who reported drinking above the low limit cut off at Year 1 had 9 times the odds of being above low levels when they progressed to Year 2 (longitudinal sample odds ratio 9.42 CI 4.82, 18.41). A similar result was found when comparing students within the cross-sectional sample at Year 1 with students in Year 2 (cross-sectional sub-sample odds ratio 8.88 CI 6.20, 12.70). For the longitudinal sample the ratio increased to 14 times the odds of remaining above the low level cut off at Year 3 (odds ratio 14.47 CI 6.21, 33.74) and the comparative figure for those in the cross-sectional sub-sample was 10.46 (CI 5.12, 21.38). Of those students in the longitudinal sample who reported drinking above low levels at Year 3, 89% were also above sensible limits at Year 1 (the corresponding figure when comparing Year 3 with Year 1 students within the cross-sectional sub-sample was 88%).

### Negative impact of alcohol consumption

From the overall sample of students who reported consuming alcohol, 34% agreed that their alcohol consumption was having a negative impact on their studies, 77% of students sampled agreed that their alcohol consumption was having a negative impact on their finances, and 48% agreed that their alcohol consumption was having a negative impact on their physical health. As would be expected, these figures decreased by academic year as the proportion engaged in heavy drinking also fell.

At each time-point those students who consumed higher than low levels of alcohol had at least 3 times the odds of reporting that alcohol was having a negative impact on their studies. They were also had at least 4 times the odds of reporting a negative impact on their finances and at least 2 times the odds of reporting that alcohol was having a negative impact on their physical health compared to students who drank at low levels (see Table 3). In contrast, only 9% of students agreed that their alcohol consumption was having a negative impact on their personal life. Students who reported drinking at low levels were as likely to report a negative impact on their personal life as those who reported drinking at higher levels.

**Table 3:** The odds of negative consequences in students reporting consumption above low levels relative to students reporting low levels of consumption

		Studies		Finance		Personal		Physical Health	
		Odds ratio	(95% CI)	Odds ratio	(95% CI)	Odds ratio	(95% CI)	Odds ratio	(95% CI)
Year 1	Longitudinal	3.02	(1.54, 5.90)	6.44	(3.08, 13.49)	0.96	(0.71, 1.30)	2.49	(1.33, 4.67)
	Cross-sectional	3.33	(2.73, 4.07)	6.16	(4.91, 7.72)	1.40	(0.53, 3.68)	3.15	(2.61, 3.80)
									
Year 2	Longitudinal	3.91	(2.05, 7.45)	4.50	(2.15, 9.43)	1.75	(0.70, 4.35)	3.54	(1.86, 6.75)
	Cross-sectional	4.59	(3.65, 5.78)	6.57	(5.07, 8.51)	1.99	(1.40, 2.81)	4.37	(3.51, 5.44)
									
Year 3	Longitudinal	9.44	(4.36, 20.45)	8.08	(3.57, 18.27)	1.33	(0.49, 3.61)	3.81	(1.90, 7.64)
	Cross-sectional	3.53	(2.70, 4.61)	7.01	(5.16, 9.53)	1.61	(1.10, 2.36)	3.44	(2.67, 4.43)

Within the longitudinal sample there was a significant difference in the perceived negative impact of drinking over time on studies (F = 5.62, df = 2, 434, p < 0.01), finance (Huynh-Feldt adjustment F = 14.02, df = 1.85, 402, p < 0.001), and physical health (F = 10.76, df = 2, 428, p < 0.001), with the perceived negative impact decreasing as students progressed through their studies. There was no significant effect of time with regard to impact on personal life (F = 1.78, df = 2, 216, p = 0.17).

## Discussion

The current observation that around 90% of students consume alcohol at least weekly is in line with the rates in the general UK population [41]. The percentage of students consuming alcohol above the recommended sensible limits is broadly in line with the UK research cited earlier [8,9,24]. Importantly, the present study found a significant reduction in the number of units per week consumed over the three-year undergraduate time span, in both the longitudinal and cross-section samples. Research in the US suggests that alcohol consumption peaks at around 21 years and then declines over time [42,43]. In the present study the consumption of UK students was highest in their first year (mean age 19 years). While we do not have pre-university alcohol consumption data this timing difference is likely to be related to the differences in minimum purchase age between the two countries. Despite the apparent difference in age of peak consumption, the current results are in line with the US literature in suggesting a 'maturing out' of high levels of consumption for many students. By their final year, around 70% of students reported drinking within low levels of consumption.

The difference in outcome between the present study and, for example, those of File et al. [23] and Newbury-Birch et al. [24] could reflect changes in student drinking patterns since 1994/1995. Alternatively, the alcohol consumption of medical and/or dental undergraduates may not reflect that of the wider student population. In addition, the timing of data collection during the academic year may also influence reported alcohol consumption [15]. It should be noted that, as in the current study, Newbury-Birch et al [24] collected during the Spring while File et al.'s [23] data were collected during the week following Freshers' (introductory) week, a time when higher alcohol intake has been observed.

Despite the significant reduction across year of study, nearly a third of students were still drinking above recommended levels in Year 3 and those who reported drinking within the high risk category during year one were more likely to still be at high risk in subsequent years. Students who maintain high levels of alcohol consumption throughout their undergraduate studies might benefit from interventions to moderate this behaviour. Research from the US suggests that understanding the developmental/time trajectories of alcohol consumption is important in determining the potential health outcomes of differing patterns of consumption over time [44,45]. While understanding the 'normative' pattern of behaviour can be helpful, this approach is limited in identifying problematic consumption patterns over time. Future research within the UK would therefore benefit from attention to trajectories of student consumption and patterns of binge drinking. This information could then be used to target interventions at students most at risk of negative health consequences.

In line with previous findings, males reported drinking more units per week on average than females [8,23,24]. However, there was no significant difference in the proportion drinking at low levels (as the limits are lower for females). This implies that in terms of potential health consequences female students are drinking at levels comparable to their male peers and suggests a need for interventions for both sexes.

The current study also shows that students are aware of the negative impact that alcohol consumption has on their studies and their finances. This finding has the potential to inform future intervention strategies, as it would suggest that the negative impact of alcohol on the financial and academic aspects of university life is more salient to students and therefore they may be more receptive to interventions emphasising these areas of their lives.

In terms of strengths and limitations, the current study is the first to investigate changes in alcohol consumption in a University-wide UK student population using a longitudinal design and including participants from all Faculties. The combination of longitudinal and cross-sectional samples, the latter including many participants, was a valuable methodological feature. However, it is important to note that 1197 participants (21%) in the cross-sectional sample responded to more than one of the three surveys meaning that the data for each year were not completely independent. Although the demographic characteristics of both the longitudinal and cross-sectional samples were representative of the student population as a whole, it is possible that the behaviour of students who chose repeatedly not to respond may be different from those that engaged. In addition, participants were asked only to record the number of units of alcohol they consumed per week. Research suggests it is common for individuals to underestimate personal consumption when asked to self-report an average value using standard alcoholic units [46], so it is likely that our results underestimate the absolute level of alcohol consumption within the student population. Future research might increase the accuracy of self-reported alcohol consumption by investigating in more detail the daily and weekly consumption levels of each person. This might be achieved by asking participants to record how many of each type of alcoholic drink was consumed over the last week using a retrospective daily diary approach.

The current study did not investigate when or where drinking occurred, the frequency of alcohol free days per week, or the occurrence of heavy episodic drinking. Given the potential health implications of heavy episodic drinking [3,6] it will be necessary for future research to investigate how heavy episodic drinking changes across the undergraduate course of study. A further study limitation was the use of a single item scale to measure the perceived negative impact of alcohol. While this provided some crude baseline information future research would benefit from using a more comprehensive and standardised measure of negative consequences.

## Conclusion

In conclusion, the observation that full-time undergraduate students' consumption of alcohol declined over the three-year course of undergraduate study is of value in directing the limited resources available for preventive interventions. Introductory weeks for first year students in UK Universities often have social events associated with, or centred on, alcohol. This is often a time when campaigns for drinking in moderation are also at their peak. Later identification, e.g. in semester two, of higher level drinkers would be of current and future benefit. However, the persistence of high levels of alcohol consumption in a large proportion of students suggests the need for effective preventative and treatment interventions for all year groups. Poor physical health, financial hardship and declining academic achievement are likely indicators of this need, albeit ones not exclusively related to problems with alcohol.

## Competing interests

The authors declare that they have no competing interests.

## Authors' contributions

All authors read and approved the final manuscript. BMB managed the wider UNIQoLL project, managed the data collection, designed the current study, developed the analysis plan and lead the writing of the paper. MB designed the wider UNIQoLL project, obtained funding and assisted in writing the paper. BM and KT assisted in analysing the data and writing the paper. AJH and WBS assisted in developing the analysis plan and writing the paper.

## Pre-publication history

The pre-publication history for this paper can be accessed here:


